# Identification of Highly Conserved SARS-CoV-2 Antigenic Epitopes with Wide Coverage Using Reverse Vaccinology Approach

**DOI:** 10.3390/v13050787

**Published:** 2021-04-28

**Authors:** Yasmin Hisham, Yaqoub Ashhab, Sang-Hyun Hwang, Dong-Eun Kim

**Affiliations:** 1 Department of Bioscience and Biotechnology, Konkuk University, Seoul 05029, Korea; yasmin91h@konkuk.ac.kr; 2 Palestine-Korea Biotechnology Center, Palestine Polytechnic University, Hebron 90100, Palestine; yashhab@ppu.edu; 3 Department of Laboratory Medicine, Asan Medical Center, University of Ulsan College of Medicine, Seoul 05505, Korea

**Keywords:** SARS-CoV-2, *in silico* approach, immunoinformatics, antigens, epitope discovery, reverse vaccinology

## Abstract

One of the most effective strategies for eliminating new and emerging infectious diseases is effective immunization. The pandemic caused by severe acute respiratory syndrome coronavirus 2 (SARS-CoV-2) warrants the need for a maximum coverage vaccine. Moreover, mutations that arise within the virus have a significant impact on the vaccination strategy. Here, we built a comprehensive *in silico* workflow pipeline to identify B-cell- and T-cell-stimulating antigens of SARS-CoV-2 viral proteins. Our *in silico* reverse vaccinology (RV) approach consisted of two parts: (1) analysis of the selected viral proteins based on annotated cellular location, antigenicity, allele coverage, epitope density, and mutation density and (2) analysis of the various aspects of the epitopes, including antigenicity, allele coverage, IFN-γ induction, toxicity, host homology, and site mutational density. After performing a mutation analysis based on the contemporary mutational amino acid substitutions observed in the viral variants, 13 potential epitopes were selected as subunit vaccine candidates. Despite mutational amino acid substitutions, most epitope sequences were predicted to retain immunogenicity without toxicity and host homology. Our RV approach using an *in silico* pipeline may potentially reduce the time required for effective vaccine development and can be applicable for vaccine development for other pathogenic diseases as well.

## 1. Introduction

SARS-CoV-2 first emerged in China, specifically in Wuhan, in December 2019 [1], and spread globally to most countries in the following months [2]. The virus causes a severe disease designated as coronavirus disease 2019 (COVID-19). Although 80% of the individuals with confirmed infection show one or more mild symptoms, including fever, fatigue, muscle or body aches, headache, nausea, diarrhea, or vomiting, 1–5% of the COVID-19-positive individuals can develop severe respiratory problems. Such symptoms may lead to acute respiratory distress syndrome (ARDS) which can be a life-threatening condition in which the lungs cannot supply enough oxygen to the vital organs of the body [1,2,3].

SARS-CoV-2 belongs to the genus of *Betacoronavirus* of the *Coronaviridae* family, which also includes the severe acute respiratory syndrome coronavirus and the Middle East respiratory syndrome coronavirus. SARS-CoV-2 is an enveloped, positive-stranded RNA virus with a genome size of approximately 29.8 kb that encodes 29 different proteins [1,4,5]. The virus has four structural proteins: surface glycoprotein (spike), envelope protein (E), membrane glycoprotein (M), and nucleocapsid protein (N). Spike is a key protein on the viral surface, which mainly binds to the host cell surface protein angiotensin-converting enzyme 2 (ACE2) to mediate the entry of the virus into the target cell [6,7]. The virus has 16 non-structural proteins (nsps); the first 11 are encoded within ORF1a, while the last 5 are encoded within ORF1b. In addition to the structural and non-structural proteins, the virus contains nine accessory proteins named ORF3a, ORF3b, ORF6, ORF7a, ORF7b, ORF8, ORF9b, ORF9c, and ORF10 [5,8,9].

With the lack of effective antiviral treatments, there is an urgent need for a protective vaccine that is effective against different strains of SARS-CoV-2 and with wide immunization coverage across the world’s nations and ethnic groups. Several projects for effective vaccine development have been undertaken, and some of these vaccines are now commercially available for human administration [10,11,12,13,14,15]. Most efforts to develop vaccines against SARS-CoV-2 focus on the spike protein as the main antigenic target. Although the spike protein is a promising B-cell epitope that is expected to induce the production of neutralizing antibodies, a mixture of diverse B- and T-cell epitopes would be more effective in ensuring a robust and life-long humoral and cell-mediated immunity. The major challenge in developing such an efficient vaccine is to discover a group of appropriate B- and T-cell epitopes that can confer immunity against a wide range of viral strains. More importantly, the current emergence of SARS-CoV-2 variant mutations worldwide warrants an urgent need for the identification of appropriate B- and T-cell epitopes of the original virus as well as of the new variants of the virus.

Since discovering antigens and epitopes by an experimental approach can be very tedious, expensive, and time-consuming, using an *in silico* approach to discover novel B- and T-cell epitopes has become the preferred strategy. This approach is considered one of the most effective for discovering antigens by screening the entire microbial proteome using various prediction tools [16]. The *in silico* vaccine candidate identification approach, designated as reverse vaccinology (RV), starts with the genetic material of the selected pathogen with the subsequent performance of rational computational predictions to come up with a manageable list of targets to be validated experimentally. This approach significantly reduces the time needed to develop a vaccine and provides reasonable targets that are identified based on the selection criteria. RV has been frequently applied with success to discover vaccine candidates for various types of pathogenic microbes (mostly bacteria) [16,17,18,19,20,21]. To date, however, RV has not been rigorously applied to the analysis of viral genomes to identify potential candidates for viral vaccine development.

Although several *in silico* studies have been carried out with the aim of identifying SARS-CoV-2 epitopes, most of these studies focused on individual proteins—mainly the spike protein—or a small subset of SARS-CoV-2 proteins [22,23,24,25,26,27,28,29,30]. Although a limited number of studies have analyzed the whole proteome of SARS-CoV-2, they identified epitopes without further comprehensive workflow to generate a shortlist of the most promising epitopes [26,27,30]. Instead, a long list of potential epitopes confined only to the T-cell category is provided, requiring further analysis and validation. Furthermore, most of these reports examined the sequence of the reference SARS-CoV-2 genome without considering emerging mutational events that may cause epitope escape. This type of epitope escape may reduce the efficacy of the immune response, resulting in sub-neutralizing antibodies that can generate antibody-dependent enhancement [31,32,33].

In the present study, we propose a comprehensive reverse-vaccinology workflow that can be used to identify and shortlist potential T- and B-cell epitopes. Starting with the analysis of three aspects of viral proteins, namely cellular location, antigenicity, and epitope density, antigenic proteins were selected, among which candidate epitopes were subsequently identified. The identified candidate epitopes were further analyzed based on their predicted antigenicity, allele coverage, and induction of interferon-gamma (IFN-γ). Furthermore, mutation analysis of all variant isolates was performed to determine the conservation of the selected epitopes. Our comprehensive approach includes both horizontal (across the whole coding regions of the genome) and vertical (across many sequenced genomes) screening for B- and T-cell epitopes. Based on our results, we provide a shortlist of the candidate epitopes for designing a potential vaccine against SARS-CoV-2, suggesting the usefulness of a comprehensive RV workflow that can be applied to other pathogens as well.

## 2. Materials and Methods

### 2.1. SARS-CoV-2 Genome Data Source

The full multi-FASTA format protein sequences of SARS-CoV-2 were downloaded from the Microbial Genomes Resources at NCBI (https://www.ncbi.nlm.nih.gov/genome).

### 2.2. Antigenic Protein Prediction

Proteins were selected based on their annotated location using the Uniport database (https://www.uniprot.org/). Proteins that were annotated as viral membrane proteins were selected as prospective B-cell immunogenic targets. In contrast, proteins that were annotated as host membrane proteins were selected as prospective T-cell activators.

Prediction of the antigenicity of the selected proteins was carried out using two web tools: AntigenPro and Vaxijen v2.0 [34,35]. AntigenPro is a sequence-based antigenicity prediction tool that uses multiple representations of the primary sequence along with five machine learning algorithms (http://scratch.proteomics.ics.uci.edu/index.html). The online tool Vaxijen predicts antigenicity based on the physicochemical properties of proteins, which is independent of sequence alignment (http://www.ddg-pharmfac.net/vaxijen/VaxiJen/VaxiJen.html). The option for the selection of the target organism was set for virus with the default cutoff score of 0.4. Next, two values obtained from the two tools were averaged and normalized, in which unity was the highest possible value and zero was the lowest possible value.

### 2.3. Epitope Mapping

The Immune Epitope Database (IEDB; https://www.iedb.org) epitope-prediction tools were applied to both the B- and T-cell candidates, as previously described [26,36]. This resource site contains a set of freely available tools for epitope prediction for both T cells and B cells and is useful for designing new vaccines. Briefly, the BepiPred algorithm was used for predicting B-cell epitopes using 0.55 as the cutoff parameter, in which the epitope sequences were required to have more than 7 amino acid residues. In addition, MHC binding predictions were used for T-cell epitopes, in which a median consensus percentile cutoff ≤20 was used for CD4(+) T-cell epitopes, and a phenotypic frequency with a 6% cutoff was used for CD8(+) T-cell epitopes. In addition, allele coverage was calculated for the CD8(+) epitopes using the 12 most common alleles (HLA-A*01:01, HLA-A*02:01, HLA-A*03:01, HLA-A*11:01, HLA-A*23:01, HLA-A*24:02, HLA-B*07:02, HLA-B*08:01, HLA-B*35:01, HLA-B*40:01, HLA-B*44:02, and HLA-B*44:03). The epitope density (ED) was calculated for proteins that were selected in the previous step by dividing the number of predicted epitopes within the protein by the length of the protein (in terms of the number of amino acids); ED of a protein = No. of epitopes/length of the protein.

### 2.4. Epitope Analyses: Antigenicity, Interferon γ Induction, Toxicity, and Host Homology

The VaxiJen v2.0 was used to prioritize epitopes based on their antigenicity using virus as the target organism with a default cutoff score of 0.4, and epitopes with 0.4 or higher scores were selected for further analyses. In addition, for the T-cell epitopes, the stringency was increased for CD4(+) by reducing the consensus percentile cutoff to 10% (as the lower the value, the higher the affinity), and for the CD8(+) T-cell epitopes, allele coverage was assessed among the 12 supertypes. Furthermore, the IFNepitope online tool (http://crdd.osdd.net/raghava/ifnepitope/) was used to predict IFN-γ induction [37].

Next, the ToxinPred online tool was used to predict the potential toxicity of the selected epitopes (http://crdd.osdd.net/raghava/toxinpred/) [38]. In addition, the NCBI BLAST website was used to detect any potential homology of the selected epitopes in the human genome (https://blast.ncbi.nlm.nih.gov/Blast.cgi). These steps were performed to exclude selected epitopes that were predicted to be toxic or had host (human) homology.

Toll-like receptors (TLRs) detect conserved pathogen-associated molecular patterns (PAMPs) of pathogens including viruses [39]. Among TLRs, activation of TLR4 has been targeted in vaccine development, as its activation directs the production of inflammatory cytokines thus assuring effective immune response [40]. Thus, for vaccine efficiency validation, docking analysis of the T-cell epitopes with the Toll-like receptor-4 (TLR4) was performed using HPEPDOCK (http://huanglab.phys.hust.edu.cn/hpepdock/) [41]. TLR4 was obtained from Protein Data Bank (ID 3FXI) and a TLR4 agonist peptide; APPHALS was retrieved as a control [42].

### 2.5. Analysis of SARS-CoV-2 Mutations

Mutations in SARS-CoV-2 viral variants at the time of the analysis were collected from the CoV-GLUE resource (http://cov-glue.cvr.gla.uk) and were then categorized for further analysis [43]. This publicly accessible web server utilizes the information on SARS-CoV-2 variants obtained from GISAID (https://www.gisaid.org/) [44,45]. Mutation analysis was performed for all SARS-CoV-2 proteins to define the distribution of mutations within the viral proteome as a percentage of mutational presence. Furthermore, the mutation density (MD) for the selected viral proteins was calculated by dividing the total number of mutations found within the protein by the length of the protein (in terms of the number of amino acids); MD = No. of total mutations/length of the protein, where the values were normalized to 1 as the highest possible value and 0 as the lowest possible value.

The site mutation density within the selected epitopes (i.e., replacement frequency) was used to heighten the selection criterion in order to maximize the effectivity range of the selected epitopes. The site mutation density was calculated at each amino acid replacement within the epitope region by dividing the number of sequences at the amino acid site with 100,000 sequences (normalized per 100,000 sequences); site mutation density = (total number of sequences within the specific mutation site)/100,000. After performing the mutation analysis using the site mutation density, the selected epitopes were divided into three categories. The first category consisted of epitope sequences with sites with a mutation density of less than 0.01. The epitopes of this category were accepted. The second category consisted of epitopes that had more than 3 sites with a mutation density greater than 0.5; the epitopes of this category were excluded. The third category comprised epitopes that had 1–3 sites with a mutation density between 0.01–0.5. The epitopes of this category were considered for reanalysis. The third category was subjected to reanalysis with online tools, such as Vaxijen, ToxinPred, and BLAST, to check the effect of the mutation in the epitope sequence on the epitope’s immunogenicity, toxicity, and host homology, respectively.

### 2.6. Structure Prediction

Among the selected immunogenic proteins, ORF10, which encodes 38 amino acids with no known homolog protein, was subjected to structural and functional prediction using PredictProtein (https://predictprotein.org/) and PEP-FOLD3 (https://bioserv.rpbs.univ-paris-diderot.fr/services/PEP-FOLD3/) online tools [46,47]. PredictProtein is a sequence-based analysis tool that predicts and annotates functions based on the secondary structure of the selected protein. PEP-FOLD3 is one of the fastest *de novo* prediction and analysis tools and provides a 3D prediction of the selected proteins and short peptides. In addition, PEP-FOLD3 was used to predict the structure of epitopes from the third category, which resulted from the mutation analysis of the epitopes, to compare epitopes with or without mutations.

## 3. Results

### 3.1. Reverse-Vaccinology Workflow Applied for the Prediction of SARS-CoV-2 Antigens

**Figure 1:**
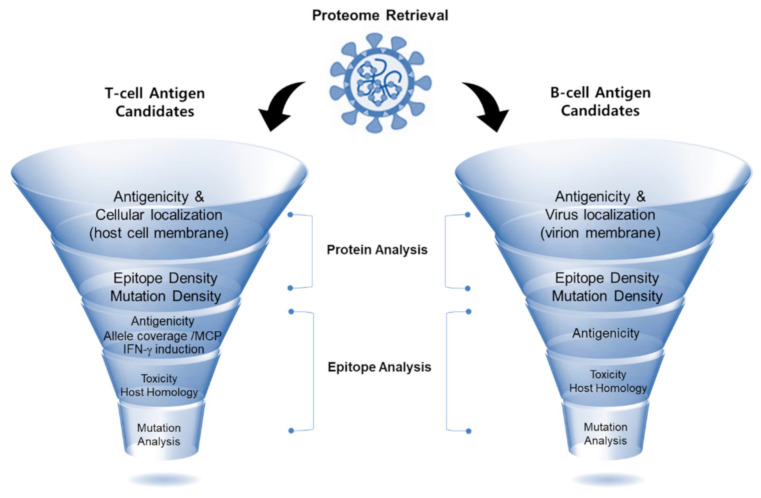
Illustration of our workflow of reverse vaccinology applied for the *in silico* screening of immunogenic SARS-CoV-2 viral epitopes. First, data retrieval of the selected pathogen (SARS-CoV-2) was carried out, which was followed by protein analysis through the determination of three aspects (cellular localization, antigenicity, and epitope density). Subsequently, the identified B-cell and T-cell epitopes were analyzed based on their predicted antigenicity, allele coverage, and IFN-γ induction (for T-cell epitopes). Next, epitopes were further checked for their toxicity and host homology. Finally, mutation analysis was conducted to determine the conservation of the selected epitopes.

Through an *in silico* approach, a comprehensive RV pipeline was proposed for better antigen identification using available online immunoinformatics tools. The *in silico* workflow of this study is illustrated in Figure 1 and consisted of two main stages of analysis: protein analysis and epitope analysis. Briefly, the proteome of SARS-CoV-2 was retrieved for the identification of both B-cell- and T-cell-candidate antigens (hereafter denoted as B-cell and T-cell antigens, respectively). The selection of antigenic protein candidates was based on the cellular location of the protein, protective antigenicity score, calculated epitope densities, and allele coverage. As for the epitope analysis, epitopes were prioritized based on their predicted antigenicity score, followed by toxicity prediction and host homology. For T-cell epitopes, the ability to induce IFN-γ and allele coverage were additionally assessed for selection. After the candidate epitopes were selected as both T-cell and B-cell antigens, mutation analysis was carried out to determine the distribution of the mutations observed in the current SARS-CoV-2 variants, within the selected proteins, as well as in the identified epitopes. All of the online analysis tools used in this study were adjusted for default parameters unless otherwise specified. Our RV-based systematic workflow was built to provide a list of the most promising candidate epitopes for the induction of B cells and T cells to augment protective immunity against the SARS-CoV-2 virus for better vaccine development.

### 3.2. Selection and Analysis of SARS-CoV-2 Proteins as Antigens Responsive to Immune Cells

Subunit vaccines provide several advantages over whole-pathogen vaccines because they are composed of parts of the pathogen, and the subunits or parts of the pathogen are sufficient to trigger and provide protective immunity (as antigens) without the need for using whole-pathogen entities. Thus, the selection of antigenic part(s) is one of the important steps in vaccine development, as it affects the effectiveness of the vaccine. Given that protein-based subunit vaccines are to be developed for immunization against the current outbreak of COVID-19, our selected proteins can be useful for the development of protein subunit-based vaccines.

In the first step, the viral proteome was examined, and candidate proteins were selected according to their annotated cellular locations based on the Uniprot database annotation. Proteins that were found on the surface of the viral membrane were considered B-cell antigens (Table 1). Three proteins were annotated as viral membrane proteins: membrane glycoprotein (M: YP_009724393.1), nucleocapsid protein (N: YP_009724397.2), and surface glycoprotein (spike: YP_009724390.1). For the T-cell antigens, five proteins were annotated as being present on the host cell membrane, and were therefore selected (Table 2): nsp3 (YP_009724389.1), nsp4 (YP_009724389.1), nsp6 (YP_009724389.1), spike (YP_009724390.1), and ORF3a protein (YP_009724391.1). Since ORF10 (YP_009725255.1) has no annotated cellular location, it was tentatively included as both a B-cell and T-cell antigen.

Protein antigenicity of the selected B-cell and T-cell antigens was predicted using both AntigenPro and VaxiJen online tools (antigenicity scores obtained from each tool are shown in Supplementary Tables S1.1 and S1.2). The average antigenicity scores for B-cell and T-cell antigens are summarized in Table 1 and Table 2, respectively. In the case of the B-cell antigens, the average protein antigenicity scores were 0.79 for the nucleocapsid phosphoprotein and 0.65 for the spike protein. Both ORF10 and membrane glycoprotein had relatively similar antigenicity scores of 0.45 and 0.42, respectively (Table 1). In case of the T-cell antigens, the average antigenicity scores were as follows: spike (0.65), ORF3a (0.50), ORF10 (0.45), nsp4 (0.42), nsp6 (0.37), and nsp3 (0.30) (Table 2). A higher antigenicity score implies a higher capability of the respective antigen to produce the desired immune response.

We noticed that the AntigenPro online tool shows different protein antigenicity scores as compared with the VaxiJen tool because they used different prediction methods (Supplementary Tables S1.1 and S1.2). The two tools gave a contradictory score for the ORF10 protein; the VaxiJen tool ranks it as the most antigenic protein on our list (0.85), while AntigenPro ranks it as the least antigenic protein (0.04). The reason why ORF10 protein gets a low score with the AntigenPro tool is likely that ORF10 protein has no similar protein reported previously. Since the AntigenPro tool is unable to analyze proteins with amino acid lengths longer than 1500, nsp3 (1944 aa) was not assessed (Supplementary Table S1.1). In contrast, the VaxiJen tool provided a score of 0.61 for nsp3, which is higher than that of the spike protein (0.55) (Supplementary Table S1.2).

After selecting the immunogenic proteins of SARS-CoV-2, 464 epitopes derived from the selected viral proteins were identified by searching the IEDB; 66 epitopes were identified as B-cell epitopes, and 398 epitopes were identified as T-cell epitopes, including 293 CD4(+) T-cell epitopes and 105 CD8(+) T-cell epitopes. Next, epitope density was calculated for each selected protein, and the values are summarized in Table 1 (for B-cell antigens) and Table 2 (for T-cell antigens). For the selected T-cell antigens, epitope density was calculated based on the activation of both CD8(+) T cells and CD4(+) T cells, along with the allele coverage for CD8(+) T cells (Table 2). The allele coverage was calculated using the following 12 alleles within the population: HLA-A*01:01, HLA-A*02:01, HLA-A*03:01, HLA-A*11:01, HLA-A*23:01, HLA-A*24:02, HLA-B*07:02, HLA-B*08:01, HLA-B*35:01, HLA-B*40:01, HLA-B*44:02, and HLA-B*44:03. The scores of allele coverage ranged from 0 to 1, in which unity corresponds to full coverage value (i.e., 12 coverages out of 12 allele subtypes).

B-cell epitope densities for three virion membrane proteins (nucleocapsid protein, membrane glycoprotein, and spike protein) were found to have similar scores at around 0.03, while ORF10 had the highest epitope density with a value of 0.131, which is 4-fold higher than that of the other three proteins. The CD4(+) epitope densities of T-cell antigens ranged from 0.015 to 0.062 (nsp6 (0.062), nsp4 (0.044), ORF3a (0.036), ORF10 (0.026), and low values for both spike protein and nsp3 (0.015 and 0.017, respectively). Thus, the spike protein and nsp3 are less likely to activate CD4(+) cells (helper T cells) because both proteins were predicted to contain fewer CD4(+) epitopes compared to the other antigenic proteins (nsp6, nsp4, ORF3a, and ORF10). In contrast, CD8(+) epitope densities ranged from 0.058 to 0.131 for the T-cell antigens; the highest value was obtained for ORF10 (0.131), followed by the nsp4 protein (0.080). Thus, ORF10 and nsp4 are very likely to activate CD8(+) cells (i.e., cytotoxic T cells). As for the allele coverage score, the lowest score (0.25) was assigned to ORF10, followed by nsp6 with a score of 0.75. The remaining three proteins (spike protein, nsp3, and nsp4) exhibited the highest score of 1.0 with maximum allele coverage, suggesting that these three proteins have a wider coverage within the population.

### 3.3. Analysis of Epitopes Selected from the Antigenic SARS-CoV-2 Proteins

Another type of subunit vaccine, the epitope-based vaccine, relies on the fundamental immunogenic components of the antigens, which are mainly responsible for protective and specific immune responses. Thus, our next step was to analyze the epitopes in our selected antigens, which were SARS-CoV-2 proteins selected in the preceding screening steps. Seven epitopes identified from the previous step using the IEDB online tool were first subjected to the VaxiJen online tool to predict their antigenicity scores with a cutoff > 0.4. Among the B-cell epitopes (Table 3), the spike protein has an epitope with a score of 1.19, and another epitope with a score of 0.88. ORF10 harbors the most antigenic epitope with a score of 1.34, while membrane glycoprotein contains an epitope with a score of 1.00 and another epitope with a score of 0.53. The two epitopes within the nucleocapsid protein have scores of 0.74 and 0.52 (Table 3).

It is well known that IFN-γ plays a crucial role in defense activation and the regulation of pathways to elicit the antiviral activity of CD8(+) and CD4(+) T cells [48,49]. In addition to the antigenicity score, the ability of the T-cell epitopes to induce IFN-γ was predicted using the IFNepitope online tool (Table 4). Subsequently, allele coverage was calculated for CD8(+) T-cell epitopes for the 12 most distributed alleles within human populations (Table 4). For the CD4(+) T-cell epitopes, allele coverage was assessed based on the IEDB consensus percentile with 10 % as a cutoff to enhance selection stringency. The IEDB consensus percentile is a uniform scale that allows comparisons between different predictors of HLA class II responses at the population level. This is a preferred method for the prediction of affinity to CD4(+) as the median consensus percentile (MCP), in which a lower value reflects a higher affinity of the epitope for CD4(+) cells. Analysis of the T-cell epitopes resulted in the identification of five epitopes for CD8(+) cells and three epitopes for CD4(+) cells, which were found to fulfill three criteria, namely antigenicity, IFN-γ induction, and allele coverage, for both CD8(+) and CD4(+) cells (Table 4). Among the T-cell epitopes, the five epitopes for the stimulation of CD8(+) cells were predicted to have maximum allele coverage (12 out of 12 allele subtypes) with different antigenicity scores. The highest antigenicity score of 1.27 was observed in the third epitope of nsp3, which was followed by the nsp6 and ORF10 epitopes with scores of 1.11 and 0.90, respectively. These three epitopes were predicted to be CD8(+) cell-activating epitopes. Moreover, with respect to the CD4(+) cell-stimulating epitopes, the ORF3a epitope had the highest antigenicity score (0.81) with a median consensus percentile of 4.8. The remaining two CD4(+) cell-stimulating epitopes, nsp3 and nsp4, with an identical score of 0.41, showed a median consensus percentile of 9.9 and 2.0, respectively. Subsequently, toxicity and host homology were assessed using ToxinPred (Supplementary Table S1.3) and BLAST-NCBI, respectively. None of the selected epitopes were predicted to have toxicity or host homology (in humans). Thus, a total of 15 epitopes in the 8 viral proteins were identified as antigenic epitopes responsive to immune cells, making them potential vaccine candidates (Table 3 and Table 4 for B-cell antigens and T-cell antigens, respectively).

Moreover, T-cell epitopes were analyzed for their TLR4 binding using the HPEPDOCK server; as a control, a synthetic lipopolysaccharide peptide was used (docking scores are summarized in Supplementary Table S2, overview of the molecular docking for epitopes is in Figure S2). Compared with the docking score of the control peptide (−152.192), all identified T-cell epitopes were predicted to have higher scores except for the epitope 1 of nsp3, TLNDLNETL, with a score of (−151.528). More importantly, the HPEPDOCK negative docking score reflects a better docking with a higher binding affinity between T-cell epitopes and TLR4, suggesting that these selected epitopes are likely promising vaccine candidates.

### 3.4. Analysis of Mutational Replacements in the Selected Epitopes

**Figure 2:**
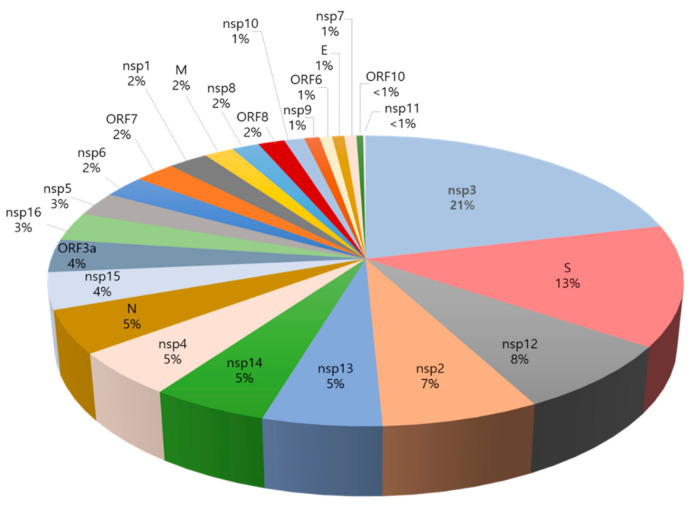
Distribution of SARS-CoV-2 mutations represented as percentage per protein (mutations collected from the CoV-GLUE).

Developing a vaccine that covers the entire population of recipients as much as possible is the most important goal in effective vaccine development. Accordingly, variations and polymorphisms in the antigens may create challenges in subunit vaccine development. Therefore, a mutation analysis of all publicly available isolates of SARS-CoV-2 derived from patients was performed to check for the mutations within the selected epitopes. First, all mutations were collected from the CoV-GLUE, a web application to track SARS-CoV-2 genome sequences (32,435 mutations at the time of analysis as of January 2021, Supplementary Table S1.4). The distribution of mutations in the SARS-CoV-2 proteome was analyzed, and the number of mutations in each protein was ranked and presented as a percentage (Figure 2). The analysis showed that 21% of mutations were distributed in the nsp3 protein followed by 13% in the spike protein. Viral proteins with a mutation distribution of less than 10% and higher than 3% were ranked in the following order: nsp12 (8%), nsp2 (7%), nsp13 (5%), nsp14 (5%), nsp4 (5%), N (5%), nsp15 (4%), and ORF3a (4%). After the analysis of the mutational distribution of the SARS-CoV-2 proteome, the mutation density (MD) for the selected proteins was calculated, in which values of MD were normalized to be 1 as the highest possible value and 0 as the lowest possible value (Table 1 and Table 2). Among our selected proteins, ORF3a has the highest mutation density (1.00) followed by the nucleocapsid phosphoprotein and nsp3 with values of 0.85 and 0.81, respectively.

Next, epitopes that were selected from our RV workflow were screened to select epitopes with minimal or null mutations (Supplementary; Table S1.5 containing the mutations among selected epitopes). To do this, the mutation density per amino acid site (i.e., site mutation density) was calculated per 100,000 sequences. The site mutation density results were grouped into three categories. The first category consisted of epitopes with mutations with MD < 0.01. These epitopes were accepted. The second category consisted of epitopes with > 3 sites, each with MD > 0.5. These epitopes were excluded. The third category consisted of epitopes with 1–3 sites, each with MD between 0.01 and 0.5. These epitopes were subjected to reanalysis.

Based on these categories, two epitopes were excluded as both harbored more than three mutations (each with MD > 0.5): nucleocapsid phosphoprotein epitopes (epitope 1: _177_RGGSQASSRSSSRSRNSSRNSTPGSSRGTSPARMAGNGG_215_ with seven mutations and epitope 2: _354_NKHIDAYKTFPPTEPKKDKKKKTDEAQPLPQRQKKQPTVTLLPAADM_400_ with five mutations). On the other hand, five epitopes were subjected to reanalysis for their antigenicity, toxicity, and host homology using new sequences with the highest frequent replacement (summarized in Table 5). Among the three epitopes of the spike protein, two epitopes were found to have mutations; the B-cell epitope (10LVSSQCVNLTTRT22) had L18F and R21I mutations, and the T-cell epitope (258WTAGAAAYY266) had a A262S mutation. The ORF10 epitope (27IAQVDVVNF35) had a V30L mutation. Comparison of the reanalyzed antigenicity scores of these epitopes to those of their original sequences showed similar and/or higher antigenicity scores. Both the membrane glycoprotein epitope (168ITVATSRTLSYYKLGASQRVAGDSGFAA195), with a T175M mutation, and the ORF3a epitope (66KKRWQLALSKGVHFV80), with a K75N mutation, showed lower antigenicity scores compared to their original sequences. Despite mutational replacements, most epitope sequences are predicted to retain immunogenicity without toxicity and host homology. Thus, filtering out 2 epitopes through mutation analysis provided a shortlist of 13 potential epitopes, from which 8 epitopes with no modification and 5 epitopes with suggested modifications are shown in Table 5.

### 3.5. Structural Prediction of Epitopes with Replacements and ORF10

**Figure 3:**
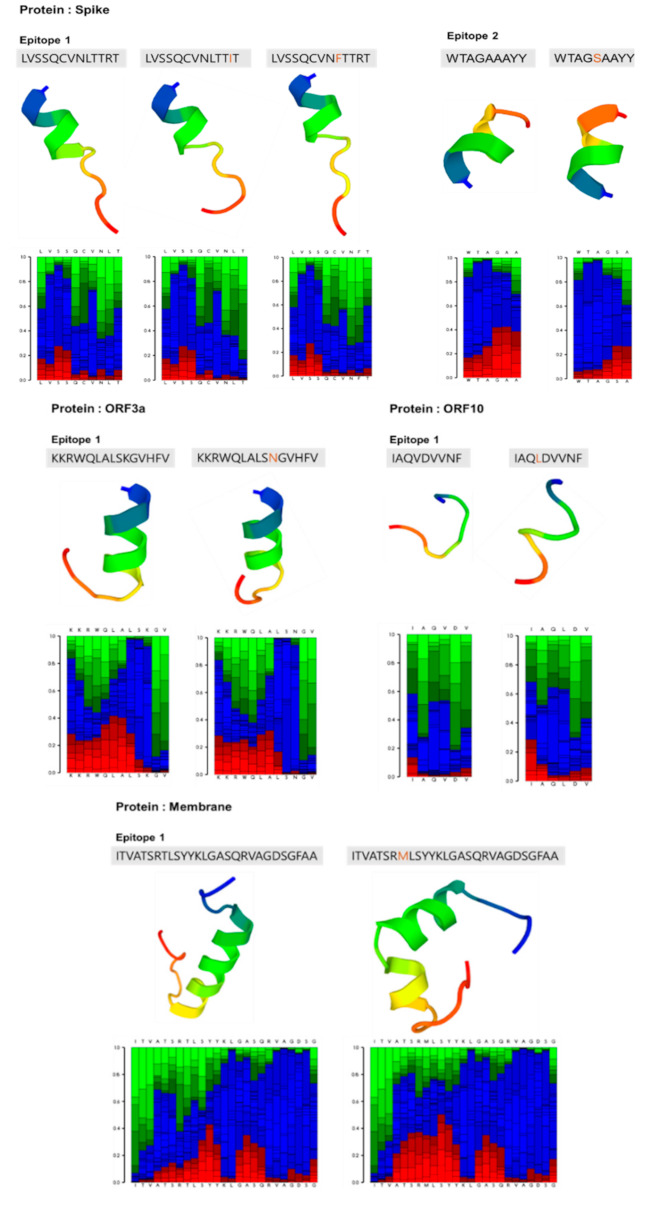
Structural prediction of epitopes with amino acid substitutions (Table 5) using the PEP-FOLD3 tool with their local structure prediction profile. Graphical presentation of local structure prediction profile with color codes: red, helical; green, extended; and blue, coil.

The structures of epitopes with replaced amino acids (Table 5) were predicted and compared with the structures of unmutated epitopes (Figure 3). As compared with the structure of the unmutated spike protein epitope 1, the mutated spike epitope 1 did not show any significant change in its alpha-helical structure with replacement of amino acids: Leu to Phe at position 18, Arg to Ile at 21, and double replacements Phe/Ile at 18/21. Similarly, the alpha-helical structures of the epitope of ORF3a and epitope 2 in the spike protein were not different from those of the unmutated epitopes. In contrast, the epitope with a replacement of T175M present in the membrane glycoprotein was predicted to exhibit a significant change in its alpha-helical structure as compared to the original epitope, which is likely to affect vaccine efficacy. Interestingly, the ORF10 epitope with the V30L mutation was predicted to have a loop structure similar to the unmutated epitope, which is likely due to its simple and random structure.

It is well known that viruses exploit the ubiquitination pathway for their pathogenesis and replication [50,51]. A previous interactome analysis of SARS-CoV-2 revealed that ORF10 protein may play a role in hijacking the ubiquitination pathway of the host proteins, in which ORF10 directly interacts with one member of the Cullin 2 (CUL2)-RING E3 ligase complex [52]. Given that ORF10 is involved in the manipulation of host proteins for viral pathogenesis, we performed a structural analysis of ORF10 despite its lack of homology to known proteins or conserved domains. The structural features of ORF10 were assessed using two protein structure prediction tools, PredictProtein and PEP-FOLD3. Several secondary structural features of the ORF10 protein were predicted (Supplementary Figure S1). Based on the ORF10 amino acid sequence, most amino acids were predicted to be exposed to the solvent (Supplementary Figure S1). Although ORF10 is a short polypeptide with 38 amino acids, it is predicted to have an alpha-helix that can form a complex with the CUL2 member. In addition, ORF10 has highly antigenic epitopes (Table 3 and Table 4), which are promising subunit vaccine candidates awaiting further validation studies in vivo. The role of ORF10 is still controversial, as there are two different views: one view relies on the lack of evidence on ORF10 expression or function in host cells, suggesting that ORF10 is a non-essential and most likely non-expressed protein [53], while the second view suggests ORF10 as a potential target for vaccine development [54], which supports our prediction.

## 4. Discussion

During the current pandemic caused by SARS-CoV-2, effective vaccination is an urgent concern. In this *in silico* study, we aimed to identify potential B- and T-cell epitopes by first analyzing probable SARS-CoV-2 proteins for a manageable list of epitopes that are promising candidates for vaccine development. Our epitope selection process involved the identification of major antigens for the stimulation of B and T cells using protein analysis and epitope-based analysis and provides a comprehensive workflow for the discovery of antigenic candidates. SARS-CoV-2 proteins, such as spike, M, and N, were selected as B-cell antigens and were reported as B-cell targets in SARS-CoV-2 patients [55]. Nucleocapsid phosphoprotein, which is responsible for the first B-cell response, has been reported to antagonize the RNAi pathway (antiviral immune defense mechanism) and play a role in immune evasion by suppressing IFN-I [56,57]. Membrane glycoprotein, which is the most abundant viral protein in SARS-CoV-2, plays a role in viral assembly and the negative regulation of the host antiviral response. It has been reported to exhibit pathogenicity through its C-terminal region [58], which was found to have a highly immunogenic domain in our study; the selected epitope (105-117) lies within the pathogenic C-terminal region (100221). The spike protein is the main target for neutralization by B cells, as it holds the receptor-binding domain (RBD) that binds to the host target cell receptor, which mediates the fusion of the viral membrane to the host cell membrane [59,60].

The alternatively selected T-cell antigens comprise non-structural proteins as potential targets, such as nsp3, nsp4, and nsp6, which together initiate and assemble the double-membrane vesicles [61,62]. The viral non-structural protein nsp3, also known as papain-like protease protein, was found to be involved in regulating the host’s innate immune response by antagonizing the NF-kB signaling and IFN induction pathways [63,64]. Nsp6 is known to be associated with the restriction of autophagosome expansion, inhibiting the transfer of viral components to lysosomes [65]. In addition, the ORF3a protein has also been selected as the T-cell antigen; this protein is known to be involved in the suppression of the innate immune response. Importantly, ORF3a is also responsible for generating cytokine storms through the activation of NF-κB and the NLRP3 inflammasome pathways [66,67].

Choosing the appropriate epitopes is the most critical step in the development of a subunit vaccine. Accordingly, our work aimed to provide a list of SARS-CoV-2 antigens consisting of viral proteins as well as epitopes. Our predicted antigens could thus be utilized to test either viral proteins to mimic their natural configuration during the development of a subunit vaccine or peptide epitopes as potent immune activator parts of the antigens during the development of peptide-based vaccines [68,69]. To gain protective antiviral immunity, both T-cell and B-cell responses were included in our study; not only were both T-cell and B-cell epitopes identified, but they were further analyzed and prioritized. Mutation analysis, host homology analysis, and epitope toxicity prediction are critical steps required to discover better vaccine candidates with higher coverage and minimal side effects.

Mutations within the selected epitopes could potentially impact the population coverage and efficiency of the vaccine. Thus, after the analysis of contemporary mutants of SARS-CoV-2 among worldwide isolates collected from the GISAID, we checked the conservation of immunogenicity in the selected epitopes to offer broader protection vaccine candidates. According to the World Health Organization, new variant strains that harbor approximately 14 mutations may impact transmissibility and therapies [70].

Among these 14 mutations, N501Y and P681H were found within the RBD of the spike protein, which was not found within the selected epitopes. In contrast, the L18F mutation, which has a high mutation frequency, is found within our selected epitopes (epitope 2 in the spike protein) [71,72]. Although L18F has a high frequency among sequences, no reported impact on virus transmissibility or severity has been reported. Hence, using this variant to design a subunit vaccine based on the respective epitopes would not hamper the coverage and immunogenic response.

Recently, a peptide microarray study of the SARS-CoV-2 proteome was performed to analyze antibody interactions at amino acid resolution [73]. Among the list of epitopes identified in the serum, several epitopes were found to be matched with our selected epitopes, such as the two epitopes derived from the membrane glycoprotein, namely _105_RTRSMWSFNPETN_117_ and _168_ITVATSRTLSYYKLGASQRVAGDSGFAA_195_, and the ORF3a-derived epitope, _66_KKRWQLALSKGVHFV_80_ (the matched sequence is underlined). In addition, an epitope derived from the spike protein—_258_WTAGAAAYY_266_—has been identified in several *in silico* studies [25,26,74,75] and was later identified as one of the epitopes that induce long-term immunity [76]. Moreover, several studies have identified that immunodominant epitopes from patients were overlapped with 8 epitopes out of 13 identified epitopes from our list [77,78,79]. Altogether, these studies further validate our approach, supporting the use of the epitopes screened in our study to develop an efficient SARS-CoV-2 vaccine. To our knowledge, our study is the first attempt to utilize a workflow involving the assessment of a series of parameters, including antigenicity, allele coverage, and mutation analysis, for evaluating the selected epitopes.

In conclusion, using an *in silico* approach to screen vaccine candidates, we obtained effective candidates that can be used for SARS-CoV-2 vaccine development. Our *in silico* RV approach was composed of two parts: (1) analysis of the various functional aspects of viral proteins, including antigenicity, allele coverage, epitope density, and mutation density, was carried out for the selected proteins based on annotated cellular location; and (2) epitopes were analyzed for antigenicity, allele coverage, IFN-γ induction, toxicity, host homology, and site mutational density. By narrowing down the list of candidate epitopes with mutational replacements, we offer a list of 13 epitopes as subunit vaccine candidates. The candidates identified using our approach can be further tested as recombinant proteins in in vitro or in vivo studies to validate their antigenicity and avoid unwanted side effects such as antibody-dependent enhancement.

## Figures and Tables

**Table 1:** B-cell antigens of SARS-CoV-2 and their corresponding antigenicity scores *, epitope density, number of mutations, and mutation density.

NCBI Ref. Seq. Accession ID	Protein Name	Length (aa)	Antigeni-city Score *	Epitope Density	No. of Mutations	Mutation Density
YP_009724393.1	Membrane glycoprotein	222	0.42	0.032	564	0.58
YP_009724397.2	Nucleocapsid phosphoprotein	419	0.79	0.031	1560	0.85
YP_009724390.1	Surface glycoprotein (spike)	1273	0.65	0.032	4360	0.78
YP_009725255.1	ORF10	38	0.45	0.131	127	0.77

* Antigenicity score was obtained by averaging two scores from both AntigenPro and VaxiJen online tools.

**Table 2:** T-cell antigens of SARS-CoV-2 and their corresponding antigenicity scores ^*^, epitope density, allele coverage, number of mutations, and mutation density.

NCBI Ref. Seq. Accession ID	Protein Name	Length (aa)	Antigen-icity Score *	Epitope Density (CD4+)	Epitope Density (CD8+)	Allele Coverage	No. of Mutations	Mutation Density
YP_009724389.1	nsp3	1944	0.30	0.017	0.065	1.00	6869	0.81
YP_009724389.1	nsp4	499	0.42	0.044	0.080	1.00	1599	0.73
YP_009724389.1	nsp6	289	0.37	0.062	0.058	0.75	801	0.63
YP_009724390.1	Surface glycoprotein (spike)	1273	0.65	0.015	0.067	1.00	4360	0.78
YP_009724391.1	ORF3a	275	0.50	0.036	0.069	0.92	1193	1.00
YP_009725255.1	ORF10	38	0.45	0.026	0.131	0.25	127	0.77

* Average values obtained from AntigenPro and VaxiJen.

**Table 3:** B-cell epitopes of SARS-CoV-2 and their corresponding antigenicity scores.

NCBI Ref. Seq. Accession ID	B-Cell Epitope Sequence	VaxiJen Score
YP_009724393.1 (M)	105 RTRSMWSFNPETN 117 (epitope 1)	1.00
	168 ITVATSRTLSYYKLGASQRVAGDSGFAA 195 (epitope 2)	0.53
YP_009724397.2 (N)	354 NKHIDAYKTFPPTEPKKDKKKKTDEAQPLPQRQKKQPTVTLLPAADM 400 (epitope 1)	0.52
	177 RGGSQASSRSSSRSRNSSRNSTPGSSRGTSPARMAGNGG 215 (epitope 2)	0.74
YP_009724390.1 (Spike)	65 FHAIHVSGTNG 75 (epitope 1)	0.88
	10 LVSSQCVNLTTRT 22 (epitope 2)	1.19
YP_009725255.1 (ORF10)	28 AQVDVVNFNLT 38	1.34

**Table 4:** T-cell epitopes of SARS-CoV-2 and their corresponding antigenicity scores, IFN-γ induction, and HLA coverage (CD8+) and/or MCP (CD4+).

NCBI Ref. Seq. Accession ID	T-Cell Epitope Sequence (Responsive T Cell)	VaxiJen Score	IFNepitope	HLA Coverage (CD8+)/ MCP*(CD4+)
(nsp3) YP_009724389.1	1437 TLNDLNETL 1445 (CD8) (epitope 1)	0.75	+	12/12
	2351 FSYFAVHFISNSWLM 2365 (CD4) (epitope 2)	0.41	+	9.9
	2901 KLIEYTDFA 2909 (CD8) (epitope 3)	1.27	+	12/12
(nsp4) YP_009724389.1	3151 KHFYWFFSNYLKRRV 3165 (CD4)	0.41	+	2.0
(nsp6) YP_009724389.1	3666 WLDMVDTSL 3674 (CD8)	1.11	+	12/12
(Spike) YP_009724390.1	258 WTAGAAAYY 266 (CD8)	0.63	+	12/12
(ORF3a) YP_009724391.1	66 KKRWQLALSKGVHFV 80 (CD4)	0.81	+	4.8
(ORF10) YP_009725255.1	27 IAQVDVVNF 35 (CD8)	0.90	+	12/12

* Median Consensus Percentile.

**Table 5:** Epitopes selected for reanalysis with their respective site mutation densities and the highest frequent replacement. New sequences of epitopes are listed with their antigenicity scores (Bold and underlined sequences denote replaced residues).

Protein Name	Site Mutation Density	Highest Frequent Replacement	New Epitope Sequence	VaxiJen Score
Spike	0.210	L18F	LVSSQCVNFTTRT (Epitope 1)	1.4
	0.016	R21I	LVSSQCVNLTTIT (Epitope 1)	1.0
	−	L18F/R21I	LVSSQCVNFTTIT (Epitope 1)	1.2
	0.024	A262S	WTAGSAAYY (Epitope 2)	0.60
M	0.012	T175M	ITVATSRMLSYYKLGASQRVAGDSGFAA	0.44
ORF3a	0.026	K75N	KKRWQLALSNGVHFV	0.70
ORF10	0.437	V30L	IAQLDVVNF	0.94

## Supplementary Materials

The following content is available online at https://www.mdpi.com/article/10.3390/v13050787/s1: Figure S1: Analysis of ORF10 protein; Figure S2: HPEPDOCK molecular docking analysis showing structures of T-cell epitopes with TLR4; Table S1, Excel spreadsheet containing the following: (1) B-cell target antigenicity scores, (2) T-cell target antigenicity scores, (3) ToxinPred results (B-cell epitopes are blue shaded, T-cell epitopes are orange shaded, and epitopes with mutations have no colored shade), (4) Mutations collected from CoV-GLUE, and (5) Mutations within the selected epitopes (replaced mutations indicated as a red-colored background; intensity indicating density); Table S2: T-cell epitopes and their docking scores.
