# Supplementary material for: Identification of Highly Conserved SARS-CoV-2 Antigenic Epitopes with Wide Coverage Using Reverse Vaccinology Approach
Source: Viruses. 2021 Apr 28;13(5):787. doi: 10.3390/v13050787 (PMC8145845; doi:10.3390/v13050787)
Supplement: Supplementary file 1 [file viruses-13-00787-s001.zip › Supplementary information_Viruses MDPI/Supplementary Material-Figure S1.pdf]

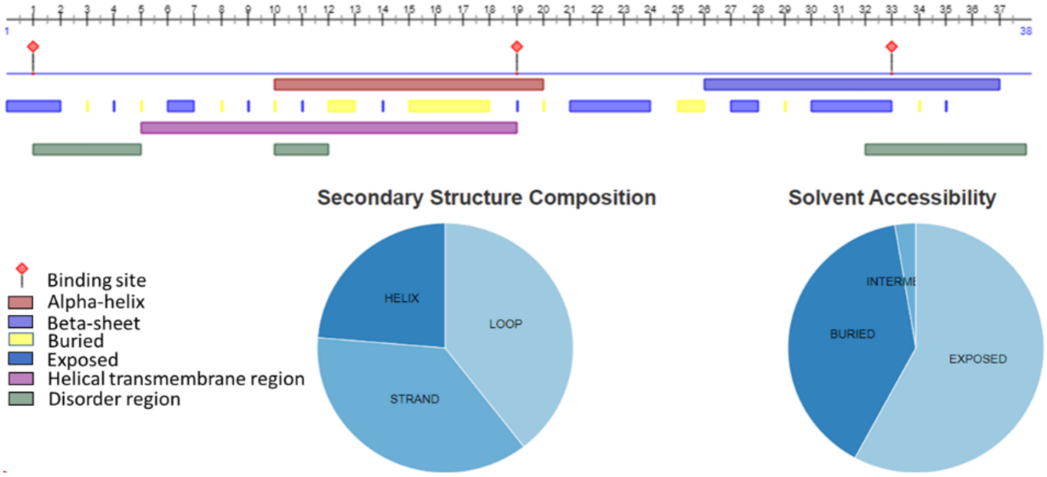

(A)

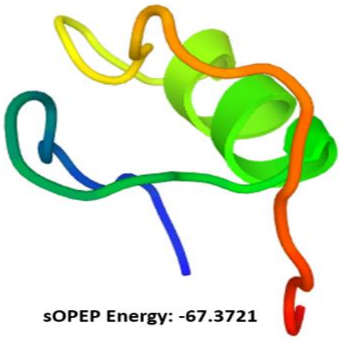

(B)

Supplementary Figure.S1 : Analysis of ORF10 protein of SARS-COV-2. A. The secondary structure and features analysis resulted from PredictProtein, and B. The predicted 3D structure resulted by PEP-FOLD3.
